# Environmental heterogeneity does not affect levels of phenotypic plasticity in natural populations of three *Drosophila* species

**DOI:** 10.1002/ece3.2904

**Published:** 2017-03-19

**Authors:** Tommaso Manenti, Jesper G. Sørensen, Volker Loeschcke

**Affiliations:** ^1^ Section for Genetics, Ecology and EvolutionDepartment of BioscienceAarhus UniversityAarhus CDenmark

**Keywords:** laboratory adaptation, local adaptation, stress resistance, temperature fluctuation

## Abstract

Adaptation of natural populations to variable environmental conditions may occur by changes in trait means and/or in the levels of plasticity. Theory predicts that environmental heterogeneity favors plasticity of adaptive traits. Here we investigated the performance in several traits of three sympatric *Drosophila* species freshly collected in two environments that differ in the heterogeneity of environmental conditions. Differences in trait means within species were found in several traits, indicating that populations differed in their evolutionary response to the environmental conditions of their origin. Different species showed distinct adaptation with a very different role of plasticity across species for coping with environmental changes. However, geographically distinct populations of the same species generally displayed the same levels of plasticity as induced by fluctuating thermal regimes. This indicates a weak and trait‐specific effect of environmental heterogeneity on plasticity. Furthermore, similar levels of plasticity were found in a laboratory‐adapted population of *Drosophila melanogaster* with a common geographic origin but adapted to the laboratory conditions for more than 100 generations. Thus, this study does not confirm theoretical predictions on the degree of adaptive plasticity among populations in relation to environmental heterogeneity but shows a very distinct role of species‐specific plasticity.

## 1 Introduction

The adaptation of populations is continuously challenged by changes in the environmental conditions (Hoffmann, Sørensen, & Loeschcke, 2003). Within generations, populations can cope with environmental changes via phenotypic plasticity (Lande, 2009; Pigliucci, 2001) where the same genotype produces different phenotypes in response to different environments (Bradshaw, 1965). Phenotypes are often strongly affected by plasticity, suggesting that plasticity plays a crucial role for the ecological and evolutionary success of populations in a changing environment. The detection of genetic variation for plastic responses (Scheiner, 1993), as well as variation in plasticity within and between species (Gibert et al., 2004; van Heerwaarden & Sgrò, 2011), suggests that plasticity itself can evolve and contribute to adaptation, for example, to changing climatic conditions (Hoffmann & Parsons, 1989; Lande, 2009). Theory predicts that several conditions are required for adaptive plasticity to evolve in a given environment in addition to genetic variance for plasticity (Agrawal, 2001; Angilletta, 2009; de Jong, 2005). First, populations should experience spatial and/or temporal heterogeneity of environmental conditions (e.g., through within generation fluctuations, Ketola & Saarinen, 2015); second, the environment should provide reliable cues for these environmental changes.

The *latitudinal hypothesis* predicts that plasticity should increase going from the equator to the poles based on the concomitant increase in thermal seasonality (Bozinovic, Calosi, & Spicer, 2011; Chown, Gaston, & Robinson, 2004; Ghalambor, Huey, Martin, Tewksbury, & Wang, 2006). Even if variation in plasticity among populations has sometimes been found, this variation does not seem to follow the adaptive pattern suggested by the latitudinal hypothesis (Ghalambor, McKay, Carroll, & Reznick, 2007; Gunderson & Stillman, 2015). For instance, Mitchell, Sgro, and Hoffmann (2011) found no clear pattern of latitudinal variation in heat hardening response across several *Drosophila* species. Furthermore, no relationship between geographic location and thermal plasticity was found in several populations of *Drosophila simulans* collected across a latitudinal gradient across Eastern Australia (van Heerwaarden, Lee, Overgaard, & Sgrò, 2014). The lack of a latitudinal adaptive pattern in plasticity of natural populations is a general finding (Fragata et al., 2016; Gunderson & Stillman, 2015; Manenti, Loeschcke, Moghadam, & Sørensen, 2015; Sørensen, Kristensen, & Overgaard, 2016) and might stem from evolutionary constraints of plasticity (Murren et al., 2015). Alternatively, the limited ability of plasticity to evolve found in these studies can partly be a consequence of the experimental design. For example, adaptive changes in plasticity can be manifested in a specific trait or be species‐specific, resulting in unclear patterns when comparing different species and different traits. Moreover, the microclimatic conditions can vary markedly within short geographic distances, reducing or breaking down the expected correlation between latitude and environmental heterogeneity (Potter, Woods, & Pincebourde, 2013). In consequence, assuming increased environmental heterogeneity at higher latitude might lead to erroneous conclusions. Finally, complex trade‐offs between abiotic and biotic factors can differ between field and laboratory conditions, which can explain the lack of adaptive evolution of the levels of plasticity found in evolutionary studies performed in the laboratory (Cavicchi, Guerra, Giorgi, & Pezzoli, 1985; Kellermann, Hoffmann, Kristensen, Moghadam, & Loeschcke, 2015; Santos et al., 2005). This, combined with the fact that artificial selection usually is executed over relatively few generations, can limit the evolution of plasticity under laboratory conditions.

In this study, we investigated the relation between environmental heterogeneity and adaptive plasticity of natural populations of three sympatric *Drosophila* species collected in both Italy and Denmark. We collected climatic data for a year prior to collection in order to precisely quantify differences in mean and variability (heterogeneity) of several environmental parameters from the two collection sites. Analyses of the climate data showed that the collection site in Denmark represented a colder habitat with lower amplitude of daily temperature fluctuations compared to the Italian collection site, especially during autumn, winter, and spring. Given differences in the mean environmental parameters and aspects of environmental heterogeneity such as amplitude of daily temperature fluctuation, we expected different levels of plasticity between populations from the two collection sites.

Plastic responses were induced by a constant, a predictable, and an unpredictable daily fluctuating thermal regime. Fluctuating temperatures can occur at different time scales. In this study, we used daily fluctuating temperatures because day–night fluctuation of temperature is ecological relevant and consistently experienced by natural populations. Moreover, daily fluctuations of temperature are experienced by flies repeatedly throughout life, and this was shown to induce a marked plastic response in numerous traits (Manenti, Sørensen, Moghadam, & Loeschcke, 2014; Sørensen, Schou, Kristensen, & Loeschcke, 2016). Finally, the same thermal regimes were used in a recent laboratory natural selection experiment on plasticity in *D. simulans* (Manenti et al., 2015), making it possible to compare results obtained in the laboratory selection study with the results of natural adaptation investigated in this study.

Testing three sympatric species that were collected at the same time allowed us to address the relation between environmental heterogeneity and adaptive plasticity in three different genetic backgrounds. This was performed to address how and if partially independent phylogenetic lines converged in their levels of plasticity when adapting to common environmental conditions. We assayed several life history (developmental time, egg‐to‐adult viability, productivity, and wing size) and stress resistance traits (time to heat knockdown, starvation tolerance) in order to get a comprehensive understanding of how adaptation to different environmental conditions was achieved among species. To further investigate the relation between plasticity and heterogeneity of environmental conditions, the Danish freshly caught population of *Drosophila melanogaster* was compared to a population collected from the same location and maintained in the laboratory for more than 100 generations. The laboratory and natural conditions are markedly different in aspects of the environment such as temperature, light, humidity, and diet. Relevant for this study, the laboratory conditions are markedly more stable (homogenous) compared to the heterogeneous natural environment. This allowed us to test the effect of adaptation to environments with extremely contrasting heterogeneity on levels of plasticity.

## 2 Material and Methods

### 2.1 Origin of fresh field populations

Three sympatric *Drosophila* species (species), *D. melanogaster*,* D. immigrans,* and *D. hydei,* were collected in Italy and in Denmark (location) during August 2014, resulting in six experimental populations (population). The Danish populations were collected in Odder (55°56′57″N 10°12′3″E; Sørensen, Kristensen, Loeschcke, & Schou, 2015). The Italian populations were collected in Località la Casella, 50 km southwest of Bologna (44°27′16″N, 11°04′32″E). Each experimental mass‐bred population was established from around 25 isofemale lines based on field caught females. From each isofemale line, we collected five male and five female individuals resulting in a total number of 250 individuals as founders for each mass‐bred population (250 individuals per three species per two locations). Populations were maintained in plastic bottles containing 50 ml of standard *Drosophila* medium (oatmeal‐sugar‐yeast‐agar) and placed in a climate chamber at constant 23°C with a 16:8‐hr light/dark cycle for two generations before start of the experiment.

To investigate more thoroughly the relationship between environmental heterogeneity and levels of plasticity, for *D. melanogaster* we also assayed a laboratory‐adapted population with a geographically identical origin to the freshly caught Danish population. This population was founded in 2010, based on 589 wild‐caught females (Schou, Kristensen, Kellermann, Schlötterer, & Loeschcke, 2014). The laboratory population was maintained at constant 25°C for one and a half year, and from there on at constant 20°C. In total, the laboratory‐adapted population was maintained in the laboratory for approximately 100 generations. Four generations before starting with the phenotypic assays, the population was moved into a climate room at constant 23°C with a 16:8‐hr light/dark cycle.

### 2.2 Thermal regimes

For each population, we prepared vials with 40 eggs per vial (7 ml fresh medium per vial). The vials were randomly divided into three groups and allowed to develop at a constant temperature, or at a predictable or an unpredictable fluctuating thermal regime designed to mimic natural daily fluctuations. The three thermal regimes (test regime) shared the same photoperiod of a 16:8‐hr light/dark cycle as well as the mean daily temperature of 23°C, but they were different in the predictability and amplitude of daily temperature fluctuations. In the constant regime (C), the temperature was maintained unchanged at 23°C; in the predictable fluctuating regime (PF), the daily temperature varied consistently following a sinus function. The daily maximum and minimum temperatures were 28 and 13°C, respectively. The daily temperatures were also following a sinus function in the unpredictable fluctuating regime (UF), but the daily maximum and minimum temperatures were randomly determined within the limits of the PF regime (for more details on the thermal regimes, see Manenti et al., 2014).

### 2.3 Phenotypic assessments

We assayed four life history traits (developmental time, egg‐to‐adult viability, productivity, and wing size) and two stress resistance traits (starvation and heat resistance). All the traits, except developmental time and viability, were tested on the same day in all species. For all phenotypic assessments, we used 5 days ± 6‐hr‐old reproductive active female flies of *D. melanogaster* and *D. immigrans*. *Drosophila hydei* only started to lay fertilized eggs when the flies were around 12 days ± 24‐hr‐old. For this reason, the phenotypic assessments for *D. hydei* were run when the flies were 20 days old.

The levels of plasticity of each population in a given trait were estimated as difference in its performance in the three thermal regimes. We assumed that differences in the performance of individual females from the same population developed in the different thermal regimes were the results of plastic responses induced by the corresponding thermal regime.

We assayed developmental time as the time spent by individual flies to complete their development from egg to adult. The developmental time in nine vials with exactly 40 eggs each (total 360 eggs) was observed for each population in each developmental regime. Developmental time was scored every 8 hr.

The egg‐to‐adult viability was calculated as the number of individuals that completed the development out of 40 eggs. We placed bunches of exactly 40 eggs in nine different vials for each population in each developmental regime.

Early productivity was assessed as total number of adult flies produced by a single female. Upon reaching reproductive maturity, individual females were placed separately into 7‐ml vials containing standard *Drosophila* medium. Flies were allowed to lay eggs for three days in the same vial. In order to ensure females being mated, two males were added to the vial with the female. The productivity was assayed for 20 individual females for each population in each developmental regime.

Wing size was estimated as wing centroid size. The right wing of individual females was dissected and mounted on glass slides in an alcohol/glycerin (1:1) solution. Images of the wings were obtained with a Leica DFC295 camera, mounted on a Leica MZ 125 microscope. The wing size was estimated as centroid size based on 11 wing landmarks (Trotta et al., 2010). The centroid size was estimated in 20 females for each population in each developmental regime.

Starvation resistance was scored as the time that flies could tolerate food deprivation before dying. Twenty individual females for each population from each test regime were placed individually into a vial containing 2 ml of agar/water solution. The number of dead flies was scored every 8 hr.

Time to heat knockdown was used as proxy of heat resistance. It was scored as time (in minutes) before flies would fall into a state of coma and were no longer able to react to external stimuli as consequence of exposure to 37.5°C. Individual females were put in 5‐ml glass vials placed in a rack and submerged into a water tank where two heat units maintained the water temperature stable at 37.5°C. The heat resistance was tested in 16 females for each population in each test regime.

### 2.4 Environmental parameters in Italy and Denmark

Environmental parameters such as daily maximum and minimum temperatures, average daily temperature, and daily average air pressure and humidity in Italy and Denmark were provided by ILMETEO SRL and the Danish Meteorological Institute, respectively. Data were recorded from the 1st of August 2013 to 31st of July 2014, i.e. the year before the collection of the populations used in this study. The Italian weather station where the data were collected is located in Vergato (44°16′57″N, 10°53′5″E), while the Danish weather station is located in Aarhus South (56°3′25″N, 9°53′11″E). Both weather stations are within 15 km and at similar altitude above sea level as the two locations of origin of the experimental populations.

### 2.5 Statistical analysis

#### 2.5.1 Italian and Danish field populations

The results of the phenotypic assessments for all traits except egg‐to‐adult viability were analyzed by a three‐way full factorial mixed‐model analysis of variance (ANOVA) with geographic location, test regime, and species as fixed effects. Egg‐to‐adult viability was analyzed with a generalized linear model, based on a binomial distribution, with geographic location, test regime, and species as fixed effects. Three species were not enough to infer species‐specific hypotheses on adaptive strategies to different locations, but they provided reliable information on how three different genetic backgrounds had adapted to the markedly different environmental conditions at the sampling locations. Given the significant interactions between species and location as well as between species and test regime, we ran an additional two‐way full factorial model (ANOVA) separately for each species, where geographic location and test regime were treated as fixed effect. The performance of the freshly caught field and the laboratory‐adapted Danish *D. melanogaster* populations was investigated by a two‐way ANOVA, with test regime and location (laboratory considered as location) as fixed effects.

#### 2.5.2 Environmental conditions in Italy and Denmark

We investigated differences in daily maximum, minimum, and average temperatures, as well as daily amplitude of temperature fluctuations, daily humidity, and air pressure between the collection sites in Italy and Denmark. For each environmental parameter, we applied PCA and scores from the PCA were used as response variables in a multivariate analysis of variance (MANOVA) with geographic location considered as a fixed effect. The MANOVA provided an observed *F*‐ratio for each environmental parameter between the two locations. *p*‐values associated with the observed *F*‐ratio were obtained by randomly assigning the daily temperature (nested within each month) to the two locations 10,000 times and scoring the generated random *F*‐ratios. The probability associated with the observed *F*‐ratio was then calculated as (*n* + 1)/(10,000 + 1), where *n* is the number of times where the random *F*‐ratio > observed *F*‐ratio. We used R version 3.3.0 (R Core Team 2015) for all analyses.

## 3 Results

**Figure 1:**
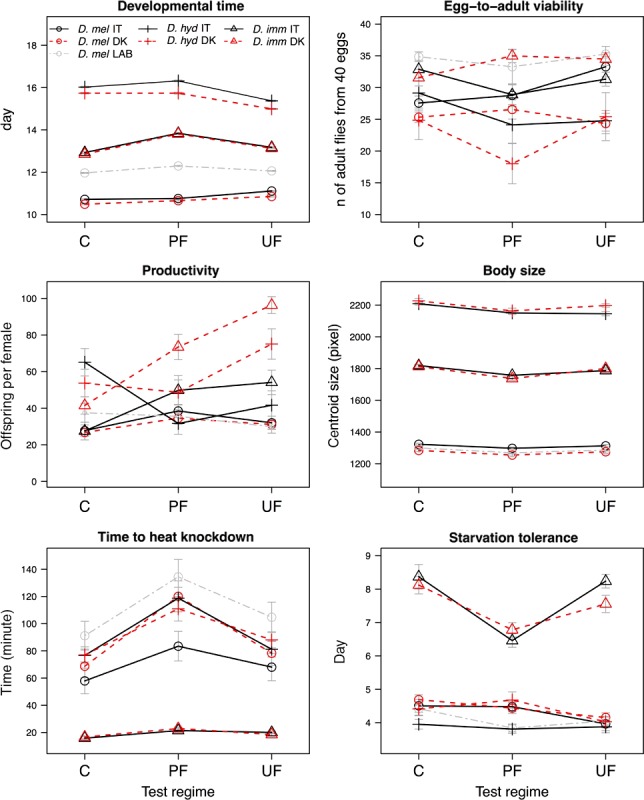
Phenotypic means of four life history and two stress resistance traits ± standard error of three *Drosophila* species from two geographic locations when tested at constant (C), predictable fluctuating (PF), and unpredictable fluctuating (UF) thermal regimes. The trait means are the response variable (*y*‐axes), while the test regime (*x*‐axes), species, and geographic locations are the explanatory variables. Open circles represent *Drosophila melanogaster*, crosses *Drosophila hydei* and open triangles stand for *Drosophila immigrans*. We marked with red color populations collected in Denmark and with black those from Italy, respectively. Gray color indicates the Danish laboratory‐adapted population of *D. melanogaster*

The three test regimes induced a plastic response in all traits investigated except in egg‐to‐adult viability (Table 1). Differences between Italian and Danish populations were observed in all traits investigated, indicating that adaption to the conditions of the collection sites had a marked effect on the performance of flies in both life history and stress resistance traits (Table 1). Significant interactions between species and test regime were found in developmental time, productivity, wing size, and starvation tolerance, indicating that different species have different levels of plasticity (Table 1, Figure 1). Each species was differently affected by the environmental conditions of origin as shown by the interaction between species and location found in all traits investigated (Table 1, Figure 1). *D. melanogaster*,* D. immigrans,* and *D. hydei* collected from Italy and Denmark were similarly affected by test regime in all traits investigated except developmental time, as indicated by the (mostly nonsignificant) three‐way interactions. In other words, the two different geographic populations of the different species all harbored the same levels of plasticity.

**Table 1:** Effects of test regime (test), geographic location (loc), species, and their interaction on different traits

Source of variation	Dev. time		Viability		Productivity		Wing size		Heat knockdown		Starvation	
	*F*	*p*	χ^2^	*p*	*F*	*p*	*F*	*p*	*F*	*p*	*F*	*p*
Test (2)	255	<.001	3.3	.19	10.7	<.001	41.1	<.001	11.7	<.001	29.9	<.001
Loc (1)	180	<.001	5.9	.01	14.8	<.001	8.58	<.01	4.1	.02	4.5	.01
Species (2)	34115	<.001	43.7	<.001	56.8	<.001	15002	<.001	403	<.001	422	<.001
Test × loc (2)	0.4	.66	0.3	.87	2.2	.11	2.1	0.12	0.2	.86	1.9	.11
Test × species (4)	278	<.001	6.5	.16	12.7	<.001	2.8	.03	0.6	.66	7.7	<.001
Loc × species (2)	30	<.001	8.3	.01	9.9	<.001	23.9	<.001	4.3	.02	3.9	.02
Test × loc × species (4)	5.5	<.001	6.1	.18	1	.40	0.2	.94	0.9	.47	1.2	.30

For all traits except viability, we used three‐way full factorial analysis of variance models, with geographic location, test regime, and species as fixed effects. Egg‐to‐adult viability was analyzed with a generalized linear model, based on a binomial distribution. The degrees of freedom are given within parentheses. The table shows the *F* (*F*) and the likelihood ratio chi‐square (χ^2^) value with associated *p*‐values (*p*) for all traits and for each parameter. Test regimes are constant, predictable, and unpredictable fluctuating thermal regimes; geographic locations are Italy and Denmark.

Looking at each species separately, we found that test regime induced a plastic response in *D. melanogaster* and in *D. immigrans* in all traits except egg‐to‐adult viability, while a plastic response was found in *D. hydei* in all traits except starvation tolerance (Table 2). The Danish population of *D. melanogaster* had shorter developmental time, lower egg‐to‐adult viability, smaller wing size, higher heat resistance, and starvation tolerance compared to the Italian population (Table 2, Figure 1). *Drosophila immigrans* collected in Italy showed a longer developmental time, lower egg‐to‐adult viability, and productivity compared to the Danish population, while no differences were found in wing size, heat knockdown time, or starvation tolerance (Table 2, Figure 1). The Danish population of *D. hydei* had a longer developmental time, lower egg‐to‐adult viability, larger wing size, and productivity and higher starvation tolerance compared to the Italian population. No difference between the Italian and Danish population of *D. hydei* was found in time to heat knockdown (Table 2, Figure 1). We found a significant interaction between location and test regime only in egg‐to‐adult viability in *D. melanogaster* and in developmental time in *D. hydei* (Table 2), confirming that overall the Italian and Danish populations of the three different species harbored similar levels of plasticity.

**Table 2:** Effects of test regime, geographic location, and their interaction on different traits in *Drosophila melanogaster*,* Drosophila immigrans*, and *Drosophila hydei*

Trait	Source of variation	*D. melanogaster*		*D. immigrans*		*D. hydei*	
		*F*/χ^2^	*p*	*F*/χ^2^	*p*	*F*/χ^2^	*p*
Dev. time	Test (2)	103	<.001	484	<.001	255	<.001
	Location (1)	73	<.001	4.8	.02	159	<.001
	Test × location (2)	4.2	.1	0.3	.75	6.7	<.01
Viability	Test (2)	1.9	.38	3.4	.18	26.4	<.001
	Location (1)	21.8	<.001	4.8	.02	18.1	<.001
	Test × location (2)	10.9	<.01	5.4	.07	4.1	.12
Productivity	Test (2)	3.7	.03	23.3	<.001	8.5	<.001
	Location (1)	2.4	.12	22.3	<.001	7.5	<.01
	Test × location (2)	0.2	.82	2.5	.09	1.9	.15
Wing size	Test (2)	8.6	<.001	25.1	<.001	11	<.001
	Location (1)	43.9	<.001	0.4	.54	8.5	<.01
	Test × location (2)	0.4	.67	1.4	.24	1.5	.22
Heat knockdown	Test (2)	9.6	<.001	12.3	<.001	17.3	<.001
	Location (1)	8	<.01	0.1	.87	0.1	.86
	Test × location (2)	0.5	.60	1.4	.26	0.7	.47
Starvation	Test (2)	7.3	<.001	21.4	<.001	1.1	.34
	Location (1)	3.3	.03	0.9	.35	9.1	<.01
	Test × location (2)	1.5	.20	1.9	.16	1.7	.20

The degrees of freedom are given within parentheses. Test regimes and geographic locations were treated as fixed effects. For all traits except viability, the table shows *F*‐ratio (*F*) with associated *p*‐values (*p*). Viability was analyzed with a generalized mixed model, and for this trait, we reported the likelihood ratio chi‐square (χ^2^) value. Test regimes are constant, predictable, and unpredictable fluctuating thermal regimes; geographic locations are Italy and Denmark.

The three thermal regimes induced plastic responses in the freshly caught and laboratory‐adapted populations of *D. melanogaster* in all traits except viability and productivity (Table 3, Figure 1). These two populations showed different performance in all traits investigated except productivity (Table 3, Figure 1). The highest viability and longest developmental time were found in the laboratory‐adapted population (Figure 1). Laboratory‐adapted flies showed larger wing size and higher heat resistance compared to the freshly caught flies (Figure 1). The same levels of plasticity were found between the Danish freshly caught and laboratory‐adapted populations of *D. melanogaster* (Figure 1) in all traits investigated except developmental time, where we observed a lower level of plasticity in the laboratory‐adapted population.

**Table 3:** Effects of test regime, geographic location, and their interaction on different traits in two populations of *Drosophila melanogaster*

Source of variation	Test (2)		Location (1)		Loc × test (2)	
	*F*/χ^2^	*p*	*F*/χ^2^	*p*	*F*/χ^2^	*p*
Developmental time	23.6	<.001	1,068.4	<.001	14.5	<.001
Viability	0.2	.90	131.1	<.001	5.4	.07
Productivity	0.6	.58	3.5	.06	1.6	.21
Wing size	12.4	<.001	4.8	.03	0.6	.57
Heat knockdown	10.8	<.001	5.9	.01	0.2	.84
Starvation resistance	5.4	<.01	6.7	.01	1.3	.27

The degrees of freedom are given within parentheses. Test regimes and geographic locations were treated as fixed effects. For all traits except viability, the table shows *F*‐ratio (*F*) with associated *p*‐values (*p*). Viability was analyzed with a generalized mixed model, and for this trait, we reported the likelihood ratio chi‐square (χ^2^) value. Test regimes are constant, predictable, and unpredictable fluctuating thermal regimes; geographic locations are Italy and Denmark.

### 3.1 Environmental conditions in Italy and Denmark

**Figure 2:**
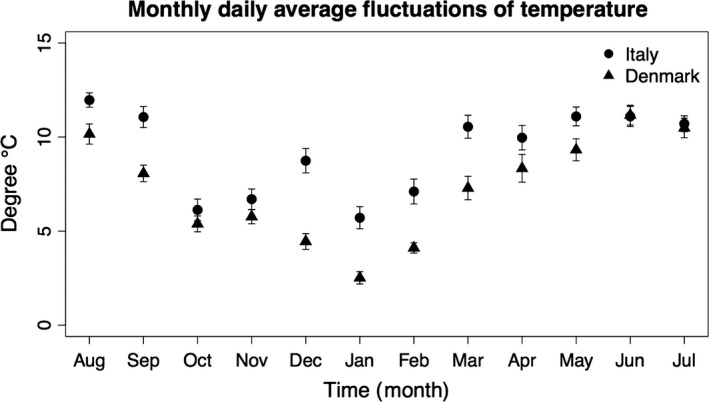
Mean (±standard error) of the daily amplitude of temperature fluctuations of the two collection sites over 12 months (from August 2013 to July 2014). Daily amplitude of temperature was calculated as difference between daily maximum and minimum temperature. Filled circles and triangles indicate the Italian and Danish collection site, respectively

The daily average and maximum temperatures were higher in Italy than in Denmark (Table 4), while lower minimum daily temperatures were recorded at the latter geographic location (Table 4). Italy showed higher amplitude of daily fluctuations and lower humidity than Denmark, while no significant differences were found in air pressure between the two geographic locations (Table 4, Figure 2).

**Table 4:** Differences between two geographic locations (Italy and Denmark) in several environmental parameters

Environmental parameters	Pillai	*F*	*p*
Maximum temperature	0.98	152.2	<.001
Minimum temperature	0.97	117	<.001
Average temperature	0.99	296	<.001
Humidity	0.96	60.3	<.001
Air pressure	0.86	15.5	.09
Amplitude of daily fluctuation of temperature	0.76	67.4	<.001

For each parameter, we applied principal component analysis on the daily scores and we used the loadings of each principal component as response variable in the MANOVA. The two geographic locations were treated as fixed effects in the model. The MANOVA provided estimates of Pillai's trace (Pillai) and approximated *F*‐ratio (*F*). Probability (*p*) associated with this *F*‐ratio was obtained by a randomization process. Degrees of freedom is 1 for the Pillai's trace. The degrees of freedom associated with *F* are 12 and 43.

## 4 Discussion

When tested in the three thermal regimes, *D. melanogaster*,* D. immigrans*, and *D. hydei* consistently expressed phenotypic plasticity in all traits except egg‐to‐adult viability. However, the levels of plasticity were found to be markedly different among species and among all traits except egg‐to‐adult viability and time to heat knockdown. Furthermore, the three species showed dissimilar patterns of plasticity in different traits. Thus, each species seems to have a unique adaptation where plasticity plays a different role for coping with environmental changes (Gibert et al., 2004). This is supported by a significant interaction between species and test regime observed in all traits investigated (Table 1). For example, the Italian and Danish populations of *D. immigrans* were more similar to each other with respect to trait means*,* and generally, with higher levels of plasticity compared to the geographic populations of the other two species. The results indicate that *D. immigrans* is able to plastically adjust its phenotype to a broad range of environmental conditions, explaining the observed lack of adaptation through changes in trait means. In contrast, the different selective pressure in Italy and Denmark affected the trait means of the other two species in several traits, suggesting that adaptation in these two species relies more on changes in trait means and less on plasticity, at least in the traits measured. Different adaptive strategies in different species might confound studies comparing evolutionary patterns of plasticity among species, contributing to the absence of the expected relationship between trait‐specific levels of plasticity and environmental conditions (Gunderson & Stillman, 2015; Mitchell et al., 2011; Sørensen, Kristensen, et al., 2016).

We found marked differences in mean performance between Italian and Danish populations in all traits investigated. This suggests that populations of the same species from the two geographic locations were adapted to the environmental conditions at their origin. We found higher heat resistance in the Danish compared to the Italian population of *D. melanogaster*. Flies selected in a warmer environment are expected to have higher heat tolerance compared to flies from a colder environment (Levins, 1969; Sørensen, Michalak, Justesen, & Loeschcke, 1999). Thus, different annual mean temperature at the collection sites was not the main driver of thermal adaptation. Furthermore, the climatic data showed that the collection site in Denmark had lower amplitude of daily temperatures compared to the collection site in Italy, especially during the autumn, winter, and spring (Table 4, Figure 2). Our results support the idea that environmental parameters, such as the amplitude of temperature fluctuations (Fallis, Fanara, & Morgan, 2011; Oliver & Palumbi, 2011) and temperature predictability (Canale & Henry, 2010; Manenti et al., 2014; Reed, Waples, Schindler, Hard, & Kinnison, 2010), rather than mean temperatures are important yet less well‐understood drivers of natural thermal adaptation.

The collection sites were markedly different in several environmental parameters, including aspects of heterogeneity such as higher amplitude of daily temperature fluctuations in Italy compared to Denmark (Table 4). Based on theory (Agrawal, 2001; Angilletta, 2009; de Jong, 2005), the levels of plasticity of the Italian populations were expected to be higher compared to the Danish populations. Our results show that different environmental conditions did not result in measurable differences in levels of plasticity between Italian and Danish populations for any trait in any species investigated, except for viability in *D. melanogaster* and for developmental time in *D. hydei*, where Danish populations had lower levels of plasticity compared to Italian ones. Accordingly, a previous study of laboratory natural selection in constant or fluctuating thermal environments in *D. simulans* found changes only in trait means, while similar levels of plasticity in the same traits were maintained in the different selection regimes (Manenti et al., 2015). The present results indicate that the low evolutionary potential of plasticity observed in the laboratory was not due to a limited number of generations of natural selection or the maintenance under laboratory conditions. The results reiterate that a change in the levels of phenotypic plasticity is not a major contributor to evolutionary adaptation within species (Fragata et al., 2016; Manenti et al., 2015) and that plasticity of environmentally adaptive traits is not consistently distributed according to simple environmental predictors such as latitude or heterogeneity (Gunderson & Stillman, 2015; Mitchell et al., 2011; Sørensen, Schou, et al., 2016).

To further investigate the effect of environmental heterogeneity, we compared trait means and levels of plasticity of the same life history and stress tolerance traits between a population adapted to the laboratory for more than 100 generations and a freshly caught Danish population of *D. melanogaster*. In line with what was found between species, similar levels of plasticity were generally found in these two populations, even though significant differences in mean performance were observed in all traits investigated except productivity (productivity, *p* = .06). Thus, adaptation to the markedly different environmental conditions did not have a major impact on the levels of plasticity. Only in developmental time, we found a difference in plasticity between the freshly caught and the laboratory‐adapted *D. melanogaster* populations*,* with a lower level of plasticity displayed by the latter population. Developmental time is strongly affected by laboratory maintenance, for example by different types of resources (Chapman & Partridge, 1996) or density (Moghadam et al., 2015). Thus, developmental time is probably one of the first targets of strong selection during laboratory adaptation. Assuming plasticity‐related costs or trade‐offs associated with high levels of plasticity, genotypes showing plasticity for developmental time were expected to be favored in natural but not in the laboratory environment. This would explain a progressive decrease in levels of plasticity for developmental time in the latter environment (Berger, Walters, & Blanckenhorn, 2014; Condon et al., 2015). Lower levels of plasticity in developmental time in the laboratory‐adapted population, as well as lower levels of plasticity in this trait in the Danish compared to the Italian freshly caught population, can be the result of these assumed trade‐offs or physiological costs associated with plasticity for this trait (Callahan, Maughan, & Steiner, 2008; DeWitt, 1998).

## 5 Conclusion

This study shows that three *Drosophila* species collected from two locations showed marked differences in the mean and plasticity of several stress resistance and life history traits. Different species had also evolved different adaptive strategies to the same environmental conditions. *D. melanogaster* and *D. hydei* rely more on adaptive changes in trait means rather than in plasticity compared to *D. immigrans*. However, contrary to the theory, but in line with what was previously found in other studies, environmental heterogeneity had a limited effect on levels of plasticity. This result was confirmed by similar levels of plasticity observed in the laboratory‐adapted and the freshly caught population of *D. melanogaster,* in all traits except developmental time. The results of this study indicate that limitations in adaptive changes in the levels of plasticity are a true feature of phenotypic plasticity.

## Conflict of Interest

The authors declare no conflict interest.

## Data Archiving

Data are archived in Dryad. doi:10.5061/dryad.q77rs.
